# Pulmonary dirofilariasis mimicking malignant pulmonary lesions: Two cases presenting as solitary and multiple pulmonary nodules

**DOI:** 10.1016/j.rmcr.2026.102523

**Published:** 2026-09-29

**Authors:** Takashi Ishihara, Tomoki Nakagawa, Haruka Kishi, Shota Fujino, Masaya Ohara, Kei Nakano, Rurika Hamanaka, Yoichiro Ikoma, Syunsuke Yamada, Ryota Masuda

**Affiliations:** a Department of General Thoracic Surgery, Tokai University, Hachioji Hospital, Japan; b Department of General Thoracic Surgery, Tokai University, Japan

## Abstract

Pulmonary dirofilariasis is a relatively rare zoonotic infection caused by *Dirofilaria immitis*, which often presents as pulmonary nodules mimicking primary lung cancer or metastatic disease on imaging. Herein, we report two cases of pulmonary dirofilariasis diagnosed using video-assisted thoracoscopic wedge resection. A man in his 60s with an 8-mm solitary pulmonary nodule and a woman in her 70s with multiple pulmonary nodules detected during follow-up after endoscopic submucosal dissection for intramucosal colorectal cancer underwent thoracoscopic wedge resection because malignancy could not be excluded. ^18^F-fluorodeoxyglucose positron emission tomography/computed tomography (FDG-PET/CT) demonstrated only mild or absent fluorodeoxyglucose uptake in the lesions. Histopathological examination revealed cross sections of *D. immitis* within the pulmonary arteries, confirming the diagnosis of pulmonary dirofilariasis in both cases. As pulmonary dirofilariasis closely resembles malignant pulmonary lesions on imaging, preoperative diagnosis remains difficult. Accordingly, this disease should be included in the differential diagnosis of solitary and multiple pulmonary nodules. Video-assisted thoracoscopic wedge resection provides a valuable approach as it provides definitive diagnosis and curative treatment.

## Highlights

• Pulmonary dirofilariasis can closely mimic malignant pulmonary nodules.
• FDG-PET/CT showed mild or absent uptake in both pulmonary lesions.
• Thoracoscopic wedge resection enabled definitive histopathological diagnosis.
• *Dirofilaria immitis* should be considered in the differential diagnosis.
• Surgical resection provides both definitive diagnosis and curative treatment.

## 1 Introduction

Pulmonary dirofilariasis (PDF) is a relatively rare zoonotic infection caused by *Dirofilaria immitis*. Humans are accidental hosts, where larvae migrate through the bloodstream and lodge in the peripheral pulmonary arteries, where they die, resulting in pulmonary infarction followed by granulomatous inflammation. Because humans are not definitive hosts, the parasite does not mature and most patients remain asymptomatic. Consequently, PDF is often detected incidentally during routine health examinations or radiological follow-up imaging of unrelated diseases.

Radiologically, PDF typically presents as a solitary subpleural pulmonary nodule that is difficult to distinguish from primary lung cancer. In contrast, the presence of multiple pulmonary nodules poses a significant clinical challenge because of the need to distinguish them from metastatic or other malignant pulmonary diseases. Although a definitive diagnosis requires histopathological identification of the parasite, preoperative diagnosis remains difficult, and many cases are diagnosed only after surgical resection.

In this report, we report two surgically diagnosed cases of PDF that mimicked malignant pulmonary lesions. One patient presented with a solitary pulmonary nodule associated with an elevated serum carcinoembryonic antigen (CEA) level, whereas the other presented with multiple pulmonary nodules during postoperative surveillance for colorectal cancer. These cases highlight that PDF should be considered in the differential diagnosis of solitary and multiple pulmonary nodules, particularly in patients with clinical findings suggestive of malignancy.

## 2 Case resentations

### 2.1 Case 1

A man in his 60s was referred to our hospital after an 8-mm pulmonary nodule was incidentally detected during a routine health examination. He had previously worked as a land surveyor. Although the patient had no history of dog ownership, he had previously kept cats.

Chest computed tomography (CT) revealed an 8-mm, well-defined, solid nodule in the subpleural region of segment 8 (S8) of the left lower lobe (Fig. 1). ^18^F-fluorodeoxyglucose (FDG) positron emission tomography/computed tomography (PET/CT) demonstrated mild uptake in the lesion (SUVmax, 1.1).

**Fig. 1:**
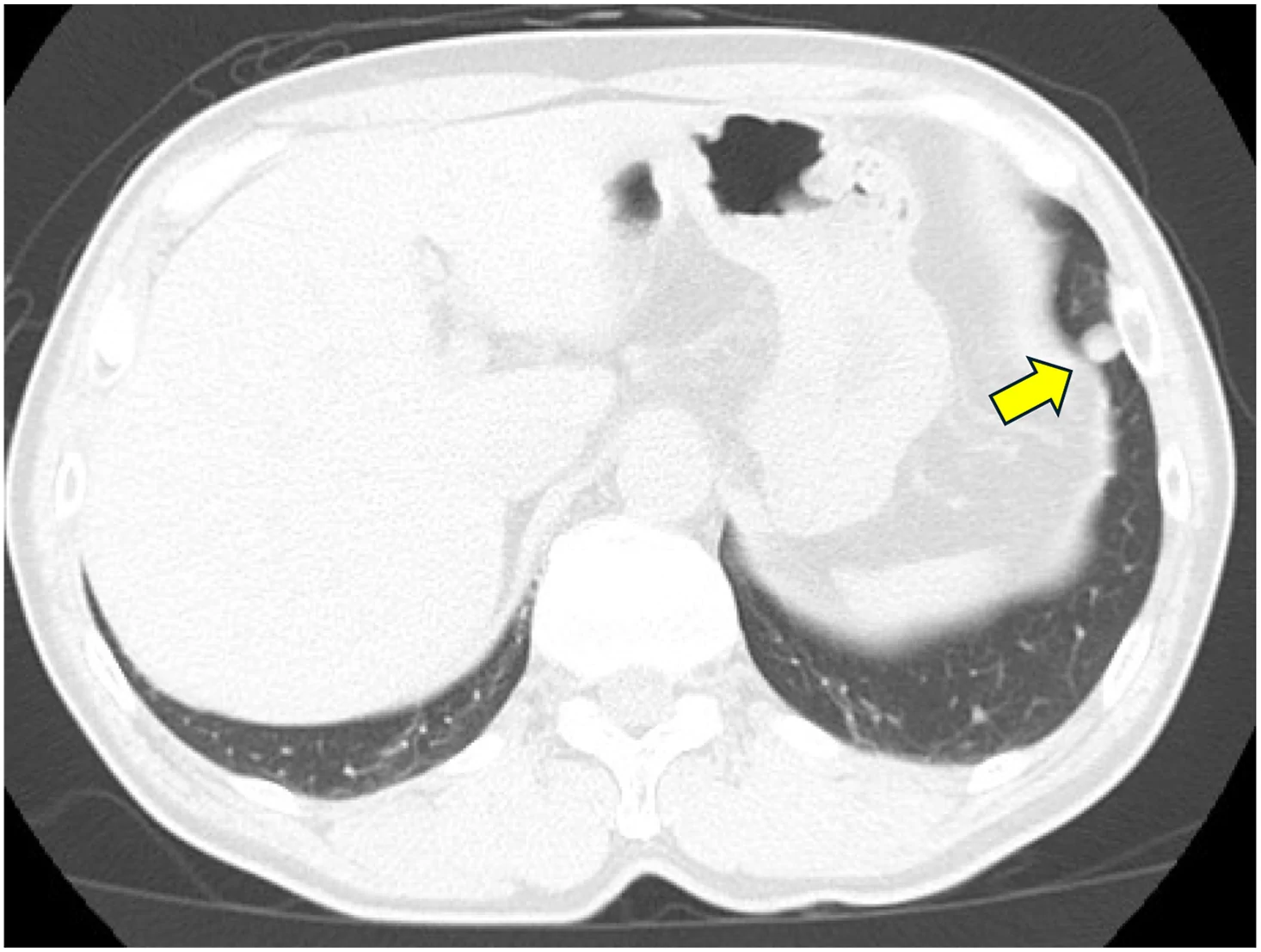
Chest computed tomography findings in Case 1 show a well-defined 8-mm solid pulmonary nodule in the subpleural region of segment 8 (S8) of the left lower lobe.

Although FDG uptake was low, the serum CEA level was markedly elevated (18.3 ng/mL), raising strong suspicion of primary lung cancer.

Video-assisted thoracoscopic wedge resection was performed to establish a definitive diagnosis. Histopathological examination revealed a degenerated cross-section of *D*. *immitis* within the peripheral pulmonary artery, which was surrounded by necrotizing granulomatous inflammation (Fig. 2). Based on these findings, the patient was diagnosed with pulmonary dirofilariasis.

**Fig. 2:**
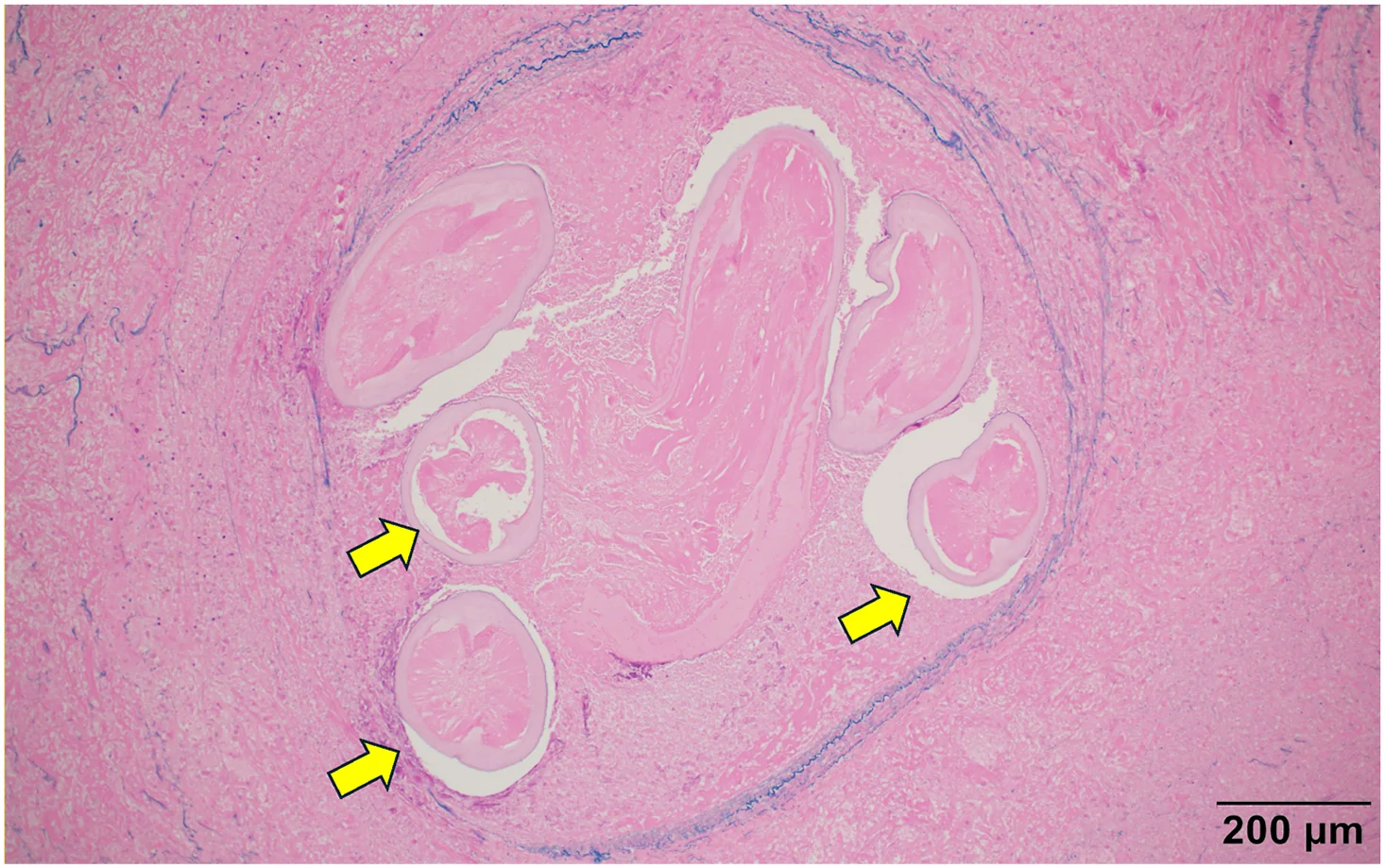
Histopathological findings in Case 1 (hematoxylin and eosin staining) show a cross-section of *Dirofilaria immitis* within the peripheral pulmonary artery surrounded by a necrotic lesion.

The postoperative course was uneventful, and no recurrence was observed during follow-up. Interestingly, the serum CEA level gradually decreased after surgery and returned to the normal range 6 years postoperatively.

### 2.2 Case 2

A woman in her 70s was referred to our hospital after two pulmonary nodules were identified during surveillance performed 9 months after endoscopic submucosal dissection for intramucosal colorectal cancer (pTisN0M0). She had kept a dog for approximately 3 years before presentation.

Chest CT demonstrated an 18-mm solid nodule in the subpleural region of segment 3 (S3) and a 10-mm subpleural nodule in segment 9 (S9) of the right lung (Fig. 3). FDG-PET/CT revealed mild uptake in the S3 lesion (SUVmax 1.0), whereas no abnormal uptake was observed in the S9 lesion.

**Fig. 3:**
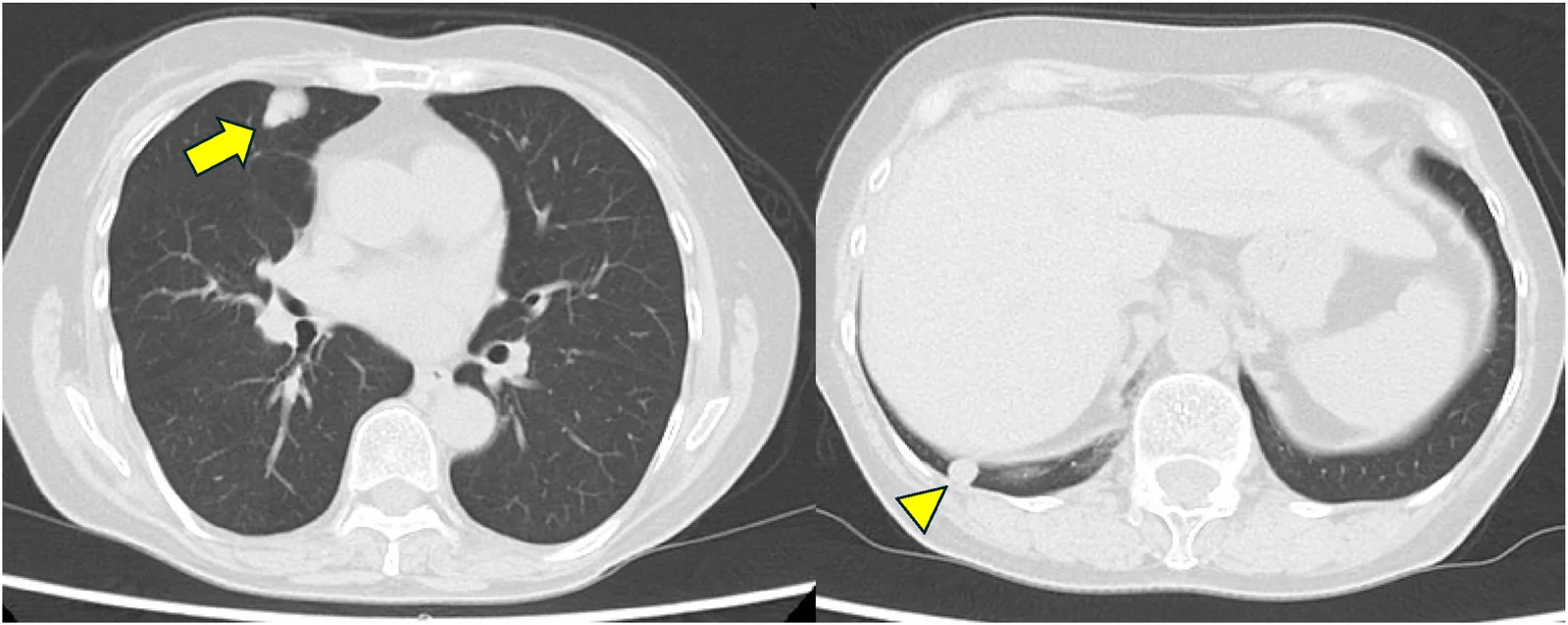
Chest computed tomography findings in Case 2 show an 18-mm solid pulmonary nodule in the subpleural region of segment 3 (S3) (arrow) and a 10-mm pulmonary nodule in the subpleural region of segment 9 (S9) (arrowhead) in the right lung.

Although pulmonary metastases were considered unlikely based on the pathological stage of the colorectal cancer, the presence of multiple pulmonary nodules made it impossible to exclude malignant disease. The serum CEA and squamous cell carcinoma antigen levels were within the normal range (3.6 ng/mL and 0.6 ng/mL, respectively).

Video-assisted thoracoscopic wedge resection was performed to establish a definitive diagnosis. Histopathological examination revealed cross-sections of *D*. *immitis* within the pulmonary arteries of both necrotic lesions, accompanied by necrotizing granulomatous inflammation (Fig. 4). Based on these findings, the patient was diagnosed with pulmonary dirofilariasis.

**Fig. 4:**
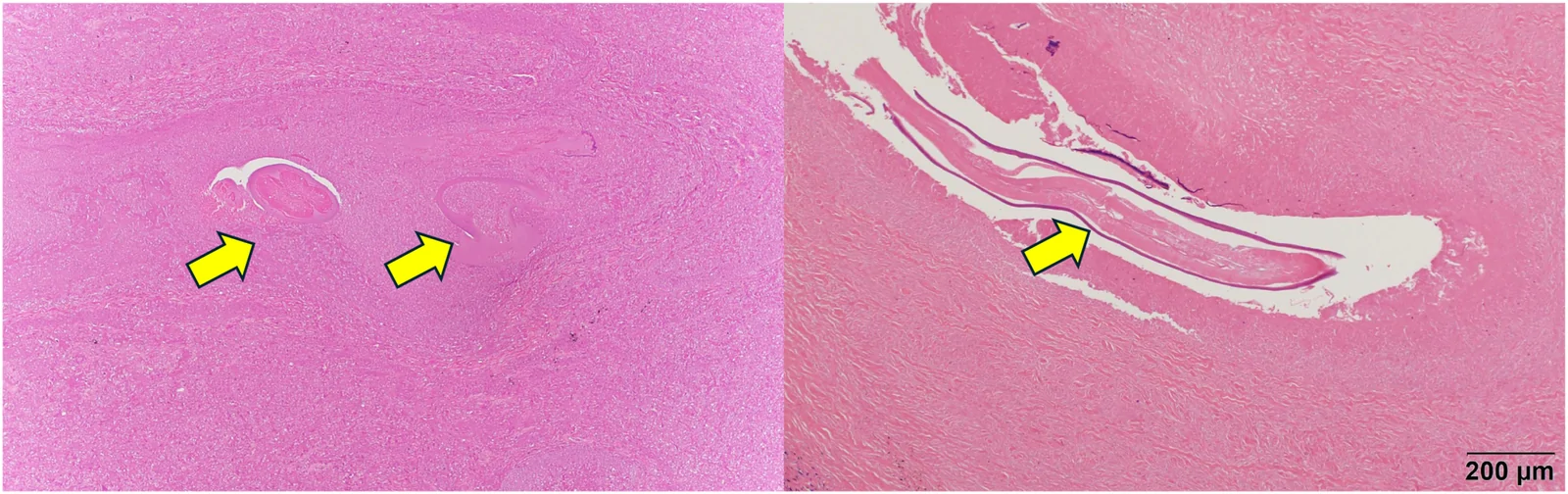
Histopathological findings in Case 2 (hematoxylin and eosin staining) show cross-sections of *Dirofilaria immitis* within a peripheral pulmonary artery in necrotic lesions of both S3 and S9.

The postoperative course was uneventful and the patient remained well without evidence of recurrence at the latest follow-up.

## 3 Discussion

PDF is an uncommon zoonotic infection caused by *D*. *immitis*. Following accidental transmission by mosquitoes, immature worms migrate to the peripheral pulmonary arteries, where they die and induce pulmonary infarction, followed by necrotizing granulomatous inflammation. Because humans are not definitive hosts, the parasite does not mature and most patients remain asymptomatic. Consequently, PDF is usually detected incidentally as pulmonary nodules on chest imaging [[1], [2], [3], [4], [5], [6]].

PDF most commonly presents as a solitary subpleural pulmonary nodule and is therefore recognized as an important differential diagnosis for primary lung cancer. Oshima et al. reviewed 69 Japanese patients and emphasized that PDF is an uncommon but clinically important cause of solitary pulmonary nodules [1]. Similarly, Ro et al. described PDF as an imitator of both primary and metastatic lung tumors, highlighting the diagnostic challenge posed by this disease [7]. Our first patient exhibited this typical presentation, with a solitary subpleural nodule that closely mimicked primary lung cancer. In contrast, our second patient presented with multiple pulmonary nodules, an uncommon presentation of PDF. Although pulmonary metastases were considered unlikely because the patient had undergone curative endoscopic submucosal dissection for intramucosal colorectal cancer (pTisN0M0), the presence of multiple pulmonary nodules made it difficult to exclude malignant disease. Human PDF presenting with multiple pulmonary nodules, has rarely been reported. Therefore, PDF should also be considered in the differential diagnosis of multiple pulmonary nodules, particularly in patients with a history of malignancy for whom metastatic disease is often the primary clinical concern.

FDG-PET/CT findings in PDF are variable, but are generally characterized by absent or only mild FDG uptake [[2], [3], [4], [5],^8^]. In our patients, the SUVmax was 1.1 in Case 1 and 1.0 in the S3 lesion of Case 2, whereas no abnormal uptake was observed in the S9 lesion of Case 2. However, low FDG uptake alone cannot reliably exclude malignancy. Surgical resection may still be required to establish a definitive diagnosis in patients with elevated tumor marker levels or a history of malignancy.

Another noteworthy finding was the markedly elevated serum CEA level in Case 1. The association between PDF and elevated serum CEA levels has not been well established. However, in Case 1, the serum CEA level gradually decreased after surgical resection and returned to the normal range at a follow-up 6 years postoperatively. Although this finding may have been coincidental, the elevated CEA level substantially increased the clinical suspicion of primary lung cancer and contributed to the diagnostic difficulty.

Histopathological identification of the parasites remains the gold standard for diagnosis. However, preoperative diagnosis is often challenging because bronchoscopic and percutaneous needle biopsies frequently fail to obtain sufficient tissue for definitive pathological evaluation. In both our patients, intraoperative frozen section examination failed to establish the diagnosis, which was confirmed only after permanent histopathological examination demonstrated cross-sections of *D*. *immitis*. Since PDF typically occurs in subpleural lesions, video-assisted thoracoscopic wedge resection is an excellent diagnostic and therapeutic approach [7,9].

Although the prevalence of *D*. *immitis* infection in dogs has declined in Japan owing to the widespread use of heartworm prophylaxis and changes in pet ownership practices, human PDF remains an important differential diagnosis for pulmonary nodules. Similar epidemiological trends have been reported in other countries. However, sporadic human infections continue to occur. Our cases demonstrate that this disease may present not only as a solitary pulmonary nodule but also as multiple pulmonary nodules mimicking malignant pulmonary lesions. Awareness of this rare entity is important because PDF may mimic both primary and metastatic lung malignancies. Recognition of this disease may facilitate accurate diagnosis while avoiding unnecessary major pulmonary resection.

## 4 Conclusion

We report two cases of PDF that mimicked malignant pulmonary lesions. The first patient presented with a solitary pulmonary nodule accompanied by an elevated serum CEA level, whereas the second patient presented with multiple pulmonary nodules during postoperative surveillance for colorectal cancer. In both cases, the lesions were located in the subpleural region and demonstrated absent or mild uptake on FDG-PET/CT; however, malignancy could not be reliably excluded based on the clinical and radiological findings alone. PDF should be considered in the differential diagnosis of solitary and multiple pulmonary nodules, particularly in patients with a history of malignancy. Video-assisted thoracoscopic wedge resection is a useful approach that enables both definitive diagnosis and curative treatment.

## CRediT authorship contribution statement

**Takashi Ishihara:** Writing – original draft. **Tomoki Nakagawa:** Writing – original draft. **Haruka Kishi:** Writing – original draft. **Shota Fujino:** Writing – original draft. **Masaya Ohara:** Writing – original draft. **Kei Nakano:** Writing – original draft. **Rurika Hamanaka:** Writing – original draft. **Yoichiro Ikoma:** Writing – original draft. **Syunsuke Yamada:** Writing – original draft. **Ryota Masuda:** Supervision.

## Consent for publication

Written informed consent was obtained from the patients and their families to publish this case report and the accompanying images.

## Data statement

All data generated or analyzed during this study are included in this article. Additional information is available from the corresponding author upon request.

## Declaration of generative AI and AI-assisted technologies in the manuscript preparation process

During the preparation of this study, the authors used ChatGPT to assist with English translation, grammar, and sentence structure. After using this tool/service, the authors reviewed and edited the content as required, and take full responsibility for the content of the published article.

## Funding

This study did not receive any specific grants from funding agencies in the public, commercial, or non-profit sectors.

## Declaration of competing interest

The authors declare that they have no known competing financial interests or personal relationships that could have appeared to influence the work reported in this paper.
